# Exploring patient preferences for intraocular lenses selection in age-related cataract: a discrete choice experiment study

**DOI:** 10.3389/fmed.2025.1446715

**Published:** 2025-02-12

**Authors:** Qiaolin Zhu, Qianqian Sun, Yujia Huo, Xiaoling Yang, Hehe Huang, Shanshan Qian, Wenjing Lin, Wentao Yan

**Affiliations:** ^1^National Clinical Research Center for Ocular Diseases, Eye Hospital, Wenzhou Medical University, Wenzhou, China; ^2^National Engineering Research Center of Ophthalmology and Optometry, Eye Hospital, Wenzhou Medical University, Wenzhou, China; ^3^State Key Laboratory of Ophthalmology, Optometry and Vision Science, Eye Hospital, Wenzhou Medical University, Wenzhou, China; ^4^Department of Gynaecological Oncology, Wenzhou Central Hospital, Wenzhou, China

**Keywords:** discrete choice experiment, cataract, health economics, patient preferences, intraocular lenses

## Abstract

**Introduction:**

This study investigates the preferences of cataract patients in East China regarding intraocular lenses (IOLs).

**Methods:**

A Discrete Choice Experiment (DCE) was conducted using a questionnaire that included various IOLs attributes. Participants made choices based on different combinations of these attributes, and the data were analyzed using multinomial logit models (MNL) and latent class analysis (LCA) to identify preference heterogeneity.

**Results:**

A total of 200 cataract patients (mean age 66.2 years, 58.5% female) participated in the study. The most influential factors in IOL selection were cost, followed by presbyopia correction, with a preference for multifocal IOLs (trifocal and bifocal), spherical aberration correction, and astigmatism correction. High cost and a higher probability of adverse visual phenomena negatively affected preferences. The inclusion of blue-blocking functionality and the surgeon’s recommendation had minimal influence on patient choice. LCA revealed three distinct preference groups: Class 1 (“Aberration Correction Seekers”) preferred aspheric IOLs, Class 2 (“Presbyopia and Blue-Blocking Enthusiasts”) favored multifocal IOLs, and Class 3 (“Astigmatism and Cost-Sensitive Patients”) preferred toric IOLs. Multinomial logistic regression analysis further showed that male patients were more likely to choose toric IOLs, while individuals with higher education levels were significantly less likely to prefer multifocal IOLs.

**Conclusion:**

This study highlights significant heterogeneity in cataract patient preferences for IOLs attributes. Cost was the most critical factor, followed by presbyopia and aberration correction. Men favored toric IOLs and exhibited sensitivity to cost, while highly educated individuals preferred multifocal IOLs less. These findings underscore the need for personalized IOLs recommendations and signal opportunities for innovation and customization in the IOLs industry.

## 1 Introduction

Cataract, characterized by lens opacification, is one of the leading causes of vision impairment, affecting approximately 95 million individuals globally. Phacoemulsification with intraocular lens (IOLs) implantation is widely regarded as the most effective and frequently performed treatment worldwide, offering significant visual rehabilitation. Advances in surgical techniques and IOLs designs over the past three decades have significantly enhanced postoperative outcomes, catering to diverse patient needs (1).

Selecting the appropriate IOLs is crucial in cataract surgery, as it directly impacts postoperative visual quality and patient satisfaction. Modern IOLs have evolved beyond simple monofocal designs to include multifocal and toric options, addressing varied refractive conditions and visual demands.

Monofocal IOLs offer clear vision at a single focal point, typically distance vision. While cost-effective, they necessitate glasses for near or intermediate activities, such as reading or computer use. Multifocal IOLs address both near and distance vision, reducing glasses dependence. However, they are associated with visual disturbances, such as glare and halos, especially under low-light conditions. Toric IOLs are tailored to correct astigmatism, providing sharper vision. Their success depends on precise alignment during surgery to achieve optimal refractive outcomes (2).

Each IOLs type has distinct strengths and limitations, necessitating a personalized approach to balance patient expectations and clinical feasibility.

Despite significant advancements in IOLs technology, research focusing on patient-specific preferences and their alignment with clinical decision-making remains limited. Traditionally, IOLs selection is guided by the surgeon’s expertise, with limited input from patients (3). However, incorporating patient preferences is essential to enhance satisfaction and surgical outcomes.

The Discrete Choice Experiment (DCE) method allows researchers to quantify patient preferences by evaluating trade-offs among IOLs attributes, providing valuable insights for personalized surgical planning (4). This study aims to utilize DCE to systematically evaluate the preferences of patients with age-related cataracts in the Wenzhou region, offering evidence-based guidance for shared decision-making in IOLs selection.

## 2 Methods

### 2.1 Discrete choice experiment

The Discrete Choice Experiment (DCE) is a widely used method in health economics and market research to quantify individual preferences for specific attributes of products or services. By presenting participants with hypothetical scenarios, DCE enables researchers to evaluate trade-offs between competing attributes and determine their relative importance. Respondents are assumed to choose the option offering the highest utility or satisfaction, reflecting the perceived value of each attribute (5).

### 2.2 Sampling

Participants were eligible if they met the following criteria:

1. Diagnosed with age-related cataract.
2. Scheduled for cataract surgery.
3. Aged between 50 and 80 years.
4. Capable of providing informed consent and willing to participate in the study.

The minimum sample size was calculated using the DCE-specific formula (6):

$n=500c/\left(t\times a\right)$

In this formula, “c” represents the highest number of levels across all attributes, “t” stands for the number of choice sets, and “a” denotes the number of alternatives within each choice set. For our specific study, this formula yielded a minimum sample size of 125, calculated as 500 × 4 / (8 × 2).

### 2.3 DCE questionnaire development

The DCE questionnaire was designed to assess key attributes and levels relevant to patient preferences for intraocular lenses (IOLs). Attributes and levels were derived from literature and clinical expert consultations, covering the following: Blue-blocking functionality, Spherical aberration correction, Astigmatism correction,

Presbyopia correction, Probability of “adverse visual phenomena, Surgeon’s recommendation, Cost (Table 1) (7–12).

**Table 1:** List of attributes and levels.

Attributes	Description	Levels
Presbyopia correction	Whether presbyopia (requiring reading glasses for clear near vision) is corrected	Trifocal (Clear vision for near, intermediate, and far distances without glasses) Bifocal (Clear vision for both near and far distances; needing glasses for intermediate) Monofocal (Clear vision for far distances; needing glasses for near and intermediate)
Spherical aberration correction	Whether spherical aberration, causing distortion in peripheral vision, is corrected.	Yes (Aspheric IOLs) No (Spherical IOLs)
Astigmatism correction	Whether astigmatism (resulting in clarity on certain axes and blurriness on others) is corrected	Yes (Toric IOLs) No (Non-toric IOLs)
Blue-blocking functionality	Whether blue light (with potential harm to the retina) is blocked	Yes No
Surgeon’s recommendation	Whether the IOL is surgeon-recommended	With Without
Cost	The expense associated with the IOL, specified in Chinese Yuan (CNY ¥)	2000, 3,000, 8,000, 10,000, 30,000
Probability of adverse visual phenomena	Risk of undesired visual effects (such as halos, glare, and starbursts) impacting post-operative vision quality	0, 10, 20, 30, 40%

Attributes often integrated in real-world IOL designs (e.g., aspheric toric lenses) were treated independently to facilitate a nuanced analysis. Using the JMP 13 Pro Choice platform, a D-efficiency fractional factorial design was applied, reducing 400 potential scenarios to two blocks of eight choice sets each. This approach optimized the questionnaire’s length while maintaining representativeness and minimizing participant fatigue.

**Figure 1:**
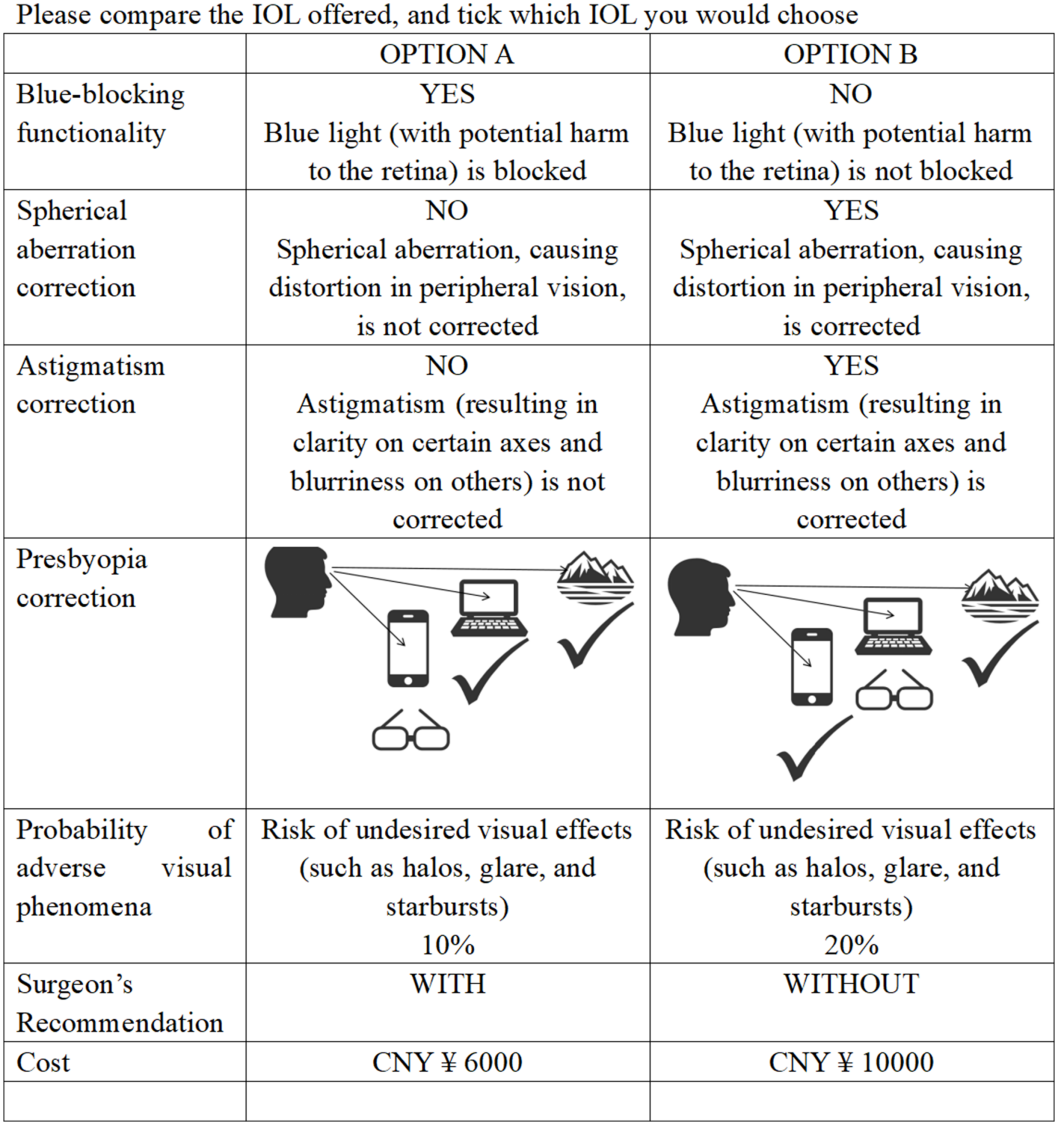
Example choice task from the discrete choice experiment.

The final questionnaire primarily consisted of two sections. The first section collected detailed demographic information, including age, gender, education level, and insurance type, as well as clinical characteristics such as laterality of cataracts, history of cataract surgery, preoperative astigmatism, glasses-wearing habits, and vision demands across near, intermediate, and far distances. The second section presented the DCE choice sets (see Figure 1 for an example of the DCE survey).

Pilot testing with 10 patients evaluated the questionnaire’s clarity and validity. To ensure response reliability, repeated tasks were included, and a dominance task validated logical preferences. Only responses passing both consistency checks were included in the final analysis. The full version of the DCE questionnaire is provided in Supplementary material S1.

### 2.4 Data collection

Data were collected between May 22 and June 15, 2023, through face-to-face interviews conducted in Mandarin or local dialects. Investigators explained the study objectives and attributes using visual aids such as diagrams and IOL models to ensure participant comprehension. Participants independently completed the questionnaire, selecting options based on personal preferences without external influence. Ethical approval was granted by the Wenzhou Medical University Affiliated Eye and Vision Hospital Ethics Committee, and the study adhered to the Declaration of Helsinki.

### 2.5 Statistical analysis

Descriptive statistics were employed to summarize patient demographics and clinical characteristics. Preferences for IOL attributes were analyzed using a multinomial logit (MNL) model, with the coefficients indicating the strength and direction of preferences. Willingness to pay (WTP) was calculated by dividing the attribute coefficients by the cost coefficient, which allowed for the determination of monetary valuations of the trade-offs. The relative importance (RI) of the attributes was calculated by dividing the range of each attribute’s coefficient across levels by the total range of coefficients for all attributes.

Latent class analysis (LCA) was performed to identify subgroups with distinct preference patterns. Models with two to seven classes were evaluated based on the Bayesian Information Criterion (BIC) and clinical interpretability. Differences between latent classes were assessed using appropriate statistical tests, including ANOVA, Chi-square, or Fisher’s exact tests. Multinomial logistic regression was conducted to identify demographic factors associated with subgroup membership.

All analyses were conducted using JMP 13 Pro Choice and Empower software. A significance level of 5% was used for all tests.

## 3 Results

### 3.1 Patient characteristics

A total of 200 participants completed the survey, with 100% (n = 200) successfully answering all DCE questions. The median time to complete the questionnaire was 3 min (range: 2–5 min). Patient characteristics are summarized in Table 2. The study population was predominantly female (58.5%), with a mean age of 66.2 ± 8.8 years. In terms of educational background, 53 participants had no formal education, 63 had completed primary school, 34 had completed junior high school, and 50 had completed high school or higher education. Regarding residence, 111 participants were from rural areas, and 89 were from urban areas. Cataract laterality was classified as unilateral (*n* = 105) or bilateral (*n* = 95). Additionally, 29 participants had previously undergone cataract surgery, while 171 had no documented history of cataract surgery.

**Table 2:** Patients’ characteristics.

Characteristic		Patients, *n* = 200
Sex	Male	83 (41%)
	Female	117 (59%)
Age	Mean ± SD years	66.5 ± 8.9 years
Education level	No formal education	53 (26.5%)
	Primary school education	63 (31.5%)
	Junior school education	34 (17%)
	High school or above	50 (25%)
Insurance	Comprehensive employment-based medical insurance	122 (61%)
	Basic medical insurance	78 (39%)
Cataract laterality	Unilateral cataracts	105 (52.5%)
	Bilateral cataracts	95 (47.5%)
History of cataract surgery	Yes	29 (14.5%)
	No	171 (85.5%)
Preoperative astigmatism	Yes	133 (66.5%)
	No	32 (16%)
	Unclear	35 (17.5%)
Glasses-wearing habits	Both	6 (3%)
	Distance only	6 (3%)
	Near only	67 (33.5%)
	Neither	88 (44%)
Near-vision demands	High	125 (62.5%)
	Moderate	75 (37.5%)
Intermediate-vision demands	High	161 (80.5%)
	Moderate	39 (19.5%)
Far-vision demands	High	166 (83%)
	Moderate	34 (17%)

### 3.2 Patient preferences

Patients showed a strong preference for presbyopia-correcting intraocular lenses (IOLs), with trifocal IOLs (coefficient = 0.353, *p* < 0.001) and bifocal IOLs (coefficient = 0.346, *p* < 0.001) significantly preferred over monofocal IOLs. Participants were willing to pay an additional CNY ¥34,962 for trifocal IOLs and CNY ¥34,723 for bifocal IOLs.

Preferences for lenses with spherical aberration correction were also evident (coefficient = 0.410, *p* < 0.001), with patients willing to pay CNY ¥27,248 for aspheric IOLs compared to spherical IOLs. Similarly, astigmatism correction was valued (coefficient = 0.234, *p* < 0.001), with participants willing to pay CNY ¥15,543 for this feature.

Regarding adverse visual phenomena, participants were willing to pay CNY ¥869.9 to reduce the risk by 1% (coefficient = −0.006, *p* = 0.025). Conversely, the inclusion of blue-blocking functionality (coefficient = 0.032, *p* = 0.377) and Surgeon’s recommendation (coefficient = 0.028, *p* = 0.377) did not significantly affect patient preferences.

Cost was identified as a significant factor, with the relative importance (RI) of cost being the highest at 35.39% (Table 3).

**Table 3:** Multinomial logit model (MNL) results and willing to pay (WTP).

Attribute	Level	Coefficients	SE	95% CI	WTP CNY ¥	*p*-value	RI
Presbyopia correction	Trifocal	0.353	0.053	0.249,0.458	34,962	<0.001	27.48%
	Bifocal	0.346	0.058	0.234,0.463	34,723		
	Monofocal	Ref.					
Spherical aberration correction	Yes (Aspheric IOLs)	0.410	0.031	0.349,0.473	27,248	<0.001	16.12%
	No (Spherical IOLs)	Ref.					
Astigmatism correction	Yes (Toric IOLs)	0.234	0.030	0.175,0.294	15,543	<0.001	9.20%
	No (Non-toric IOLs)	Ref.					
Blue-blocking functionality	Yes	0.032	0.032	−0.031,0.097	2,145	0.377	1.25%
	No	Ref.					
Surgeon’s recommendation	With	0.028	0.031	−0.033,0.090	1899	0.377	1.10%
	Without	Ref.					
Probability of adverse visual phenomena	Increase risk by 1%	−0.006	0.002	−0.012,-0.001	−869.9	0.025	9.43%
Cost	CNY ¥1 increase	−0.00003	0.000009	−0.00005, −0.00001	–	0.002	35.39%

SE, standard error; 95% CI: 95% confidence intervals; RI, relative importance; CNY ¥: Chinese Yuan; Ref., reference.

### 3.3 Probability of uptake

**Figure 2:**
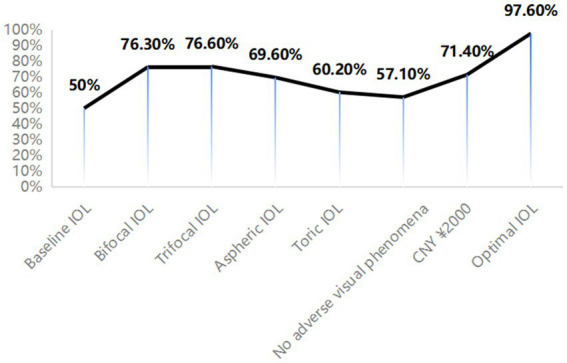
Effects of changing the attribute levels on the probability of choosing a IOLs from base uptake of 50%. The scenario from which these attribute levels were varied is: spherical design, monofocal, non-toric design, a 40% probability of adverse visual phenomena, no blue-blocking, no specific recommendation from surgeon, and a cost of CNY ¥30,000.

Figure 2 illustrates the uptake probabilities of different IOL options. The baseline IOL was defined as having a spherical design, monofocal, non-toric, a 40% probability of adverse visual phenomena, no blue-blocking, no surgeon’s recommendation, and a cost of CNY ¥30,000.

When presbyopia correction was altered from monofocal to bifocal, the uptake rate increased by 26.30%. Transitioning to trifocal IOLs resulted in a 26.60% increase in uptake. Changing the design from spherical to aspheric for spherical aberration correction increased the acceptance rate by 19.60%. The introduction of astigmatism correction from non-toric to toric resulted in an 10.20% increase in uptake. Reducing the probability of adverse visual phenomena to 0% led to a 7.10% increase in uptake. A reduction in cost to CNY ¥2,000 resulted in a 21.40% increase in uptake. The estimated uptake rate for the optimal IOLs (aspheric, trifocal, toric, 0% probability of adverse visual phenomena, CNY ¥2,000, blue-blocking, and with surgeon’s recommendation) was 97.60%.

### 3.4 Preference heterogeneity

To examine preference heterogeneity, models with two to seven classes were tested. The four-class model and beyond were deemed uninterpretable due to sample size limitations and lack of clinical relevance. Based on the Bayesian Information Criterion (BIC) and clinical interpretability, the three-class model was identified as the most suitable for describing patient preference heterogeneity. This model identified three distinct subgroups, each with unique preference patterns.

- Class 1: Aberration Correction Seekers (34% of participants) showed a strong preference for spherical aberration correction (coefficient = 0.856, *p* < 0.001), with this being the most important attribute for this group. They also valued surgeon recommendations (coefficient = 0.060, *p* = 0.035) and minimized adverse visual phenomena (coefficient = −0.001, *p* = 0.017).
- Class 2: Presbyopia and Blue-Blocking Enthusiasts (35.5% of participants) prioritized presbyopia correction, especially for trifocal (coefficient = 0.714, *p* < 0.001) and bifocal IOLs (coefficient = 0.758, *p* < 0.001). They also valued blue-blocking functionality (coefficient = 0.127, *p* < 0.001) and spherical aberration correction (coefficient = 0.093, *p* < 0.001), but were less inclined toward astigmatism correction (coefficient = −0.055, *p* = 0.003).
- Class 3: Astigmatism and Cost-Sensitive Patients (30.5% of participants) strongly preferred astigmatism correction (coefficient = 0.700, *p* < 0.001), and also valued surgeon recommendations (coefficient = 0.124, *p* < 0.001). Additionally, they showed a preference for lower-cost options (coefficient = −0.004, *p* = 0.002; Table 4).

**Table 4:** Latent class analysis with three classes.

Attribute	Level	Class 1 Coeff.	34% *p*- value	Class 2 Coeff.	35.5% *p*- value	Class 3 Coeff.	30.5% *p*- value
Presbyopia correction	Trifocal	0.001	0.956	0.714	<0.001	−0.054	0.172
	Bifocal	−0.007	0.777	0.758	<0.001	0.039	0.204
	Monofocal	Ref.		Ref.		Ref.	
Spherical aberration correction	Yes (Aspheric IOLs)	0.856	<0.001	0.093	<0.001	0.015	0.633
	No (Spherical IOLs)	Ref.		Ref.		Ref.	
Astigmatism correction	Yes (Toric IOLs)	0.046	0.112	−0.055	0.003	0.700	<0.001
	No (Non-toric IOLs)	Ref.		Ref.		Ref.	
Blue-blocking functionality	Yes	−0.004	0.860	0.127	<0.001	0.043	0.051
	No	Ref.		Ref.		Ref.	
Surgeon’s recommendation	With	0.060	0.035	−0.014	0.426	0.124	<0.001
	Without	Ref.		Ref.		Ref.	
Cost	CNY ¥1 increase	0	0.159	0.00001	0	−0.004	0.002
Probability of adverse visual phenomena	Increase risk by 1%	−0.001	0.017	0.001	0.084	−0.001	0.238

Coeff., coefficients; ref., reference.

Univariate analysis revealed no statistically significant differences across the three groups in terms of age (*F*-value = 2.399, *p* = 0.093), cataract laterality (Chi-square = 1.850, *p* = 0.396), regular glasses-wearing habits (Chi-square = 9.758, *p* = 0.135), history of cataract surgery (Chi-square = 0.274, *p* = 0.872), preoperative astigmatism (Chi-square = 3.087, *p* = 0.213), near-vision demands (Chi-square = 1.750, *p* = 0.416), intermediate-vision demands (Chi-square = 5.614, *p* = 0.061), and far-vision demands (Chi-square = 0.435, *p* = 0.804). However, significant differences were found for gender (Chi-square = 13.532, *p* = 0.001), education level (Chi-square = 30.731, *p* = 0.001), and insurance type (Chi-square = 10.664, *p* = 0.004).

Multinomial logistic regression further revealed that Class 3 (Astigmatism and Cost-Sensitive Patients) had a significantly higher proportion of males compared to Class 1 (Aberration Correction Seekers) (OR = 2.635, *p* = 0.012). Individuals with a high school education were significantly less likely to prefer IOLs with “Presbyopia and Blue-Blocking” features compared to those with “Aberration Correction” characteristics (OR = 0.264, *p* = 0.039). No significant differences were observed between the three classes regarding insurance type (*p* > 0.05; Table 5).

**Table 5:** Multinomial logistic regression outcomes: class-specific comparisons of odds ratios (95% CI) and *p*-values.

	CLASS 1	CLASS 2	CLASS 3
Intercept	1.0 (ref.)	2.052 (1.098, 3.834) 0.024	0.290 (0.109, 0.776) 0.013
Sex: Male vs. Female	1.0 (ref.)	0.881 (0.410, 1.894) 0.746	2.635 (1.237, 5.610) 0.012
Education Level: Primary School vs. No Formal Education	1.0 (ref.)	0.587 (0.235, 1.465) 0.254	1.901 (0.585, 6.177) 0.284
Education Level: Middle School vs. No Formal Education	1.0 (ref.)	0.689 (0.231, 2.058) 0.505	2.115 (0.557, 8.034) 0.271
Education Level: High School or Above vs. No Formal Education	1.0 (ref.)	0.264 (0.074, 0.937) 0.039	2.674 (0.712, 10.034) 0.144
Insurance Type: Comprehensive Employment-Based Medical Insurance vs. Basic Medical Insurance	1.0 (ref.)	0.623 (0.255, 1.523) 0.299	0.973 (0.403, 2.351) 0.952

95% CI, 95% confidence intervals; ref., reference.

## 4 Discussion

This study provides a comprehensive analysis of cataract patients’ preferences regarding IOLs attributes, marking a significant advancement in understanding patient-centered decision-making in cataract surgery. By employing a discrete choice experiment (DCE), we quantified the relative importance (RI) of various IOLs features, revealing that cost (RI: 35.39%) was the most influential factor driving patient preferences, followed by presbyopia correction (RI: 27.48%), aberration correction (RI: 16.12%), astigmatism correction (RI: 9.20%), and adverse visual phenomena (RI: 9.43%). In contrast, blue-blocking functionality and the surgeon’s recommendation had minimal impact on patient preferences.

The prominence of cost as the most important attribute (RI: 35.39%) underscores the financial burden associated with IOLs selection. In healthcare systems where premium IOLs are not fully covered by insurance, cost considerations can limit access to advanced technologies. Our findings indicate that patients are not only willing to pay more for advanced features, such as presbyopia correction and spherical aberration correction, but they also express a strong preference for minimizing overall expenses. This highlights the urgent need for healthcare policies aimed at reducing financial barriers to high-quality care. Subsidies, insurance coverage for premium IOLs, or innovative financing options could enable more patients to access advanced IOLs technologies, thereby improving both visual outcomes and quality of life. Moreover, affordability should be a central element in shared decision-making processes between surgeons and patients, ensuring that patients’ financial circumstances are considered when discussing surgical options.

### 4.1 Presbyopia correction and visual needs

Presbyopia correction emerged as a key factor in cataract patients’ preferences (RI: 27.48%), with a clear preference for multifocal IOLs, such as trifocal and bifocal lenses. These lenses address the post-surgical need for both near and distant vision, which is crucial for enhancing patients’ quality of life. This preference aligns with the growing demand for advanced IOLs options that reduce or eliminate the need for glasses, especially among patients with significant near-vision demands. The preference for multifocal lenses presents an opportunity for manufacturers to further refine their designs, offering a range of customizable options to cater to the diverse needs of cataract patients. Clinically, the results underscore the importance of considering presbyopia correction in IOLs selection, particularly for patients whose daily activities demand both near and far vision.

### 4.2 Aberration and astigmatism correction

Aberration correction (RI: 16.12%) was also a significant determinant in patient preferences. Aspheric lenses, which correct for spherical aberrations, were preferred for their ability to enhance visual quality by reducing blur and distortion, improving contrast sensitivity, and providing clearer vision for daily activities. This suggests that ophthalmologists should consider evaluating corneal spherical aberration in patients to optimize lens selection. Furthermore, astigmatism correction, particularly through the use of toric IOLs (RI: 9.20%), was another important feature, although its impact was less pronounced compared to other factors. This finding is consistent with previous research suggesting that a considerable proportion of cataract patients suffer from preoperative corneal astigmatism, making them potential candidates for toric IOLs implantation (13, 14). However, this study did not assess whether participants were suitable candidates for toric IOLs based on their ocular characteristics. Therefore, while patient preferences are essential, suitability for toric IOLs should still be determined by the surgeon based on clinical factors such as corneal condition and astigmatism severity (15).

### 4.3 Adverse visual phenomena and patient expectations

Adverse visual phenomena, such as glare or halos, were noted to influence patient preferences, though this effect was smaller compared to other attributes (RI: 9.43%). High-end IOLs, particularly multifocal and toric lenses, can lead to such phenomena, especially in low-light conditions. These effects typically diminish over time as patients adapt, but they can initially impact visual comfort, especially during activities such as night driving. It is crucial for ophthalmologists to manage patient expectations by openly discussing the potential for such side effects while emphasizing the long-term benefits. Patients should be well-informed about the nature of these phenomena and understand that they are usually transient. This approach will help mitigate concerns and ensure greater satisfaction post-surgery.

### 4.4 Blue-blocking functionality and Surgeon’s recommendation

Interestingly, blue-blocking functionality and the surgeon’s recommendation had minimal impact on patient preferences in this study. While blue-blocking lenses are marketed as offering retinal protection and improving sleep quality (16, 17), the existing evidence on these claims is mixed. Some studies suggest potential benefits, while others report negligible effects on sleep quality or visual performance (18, 19). The lack of strong preference for blue-blocking lenses in this study may be attributed to this ambiguity in the scientific literature. Additionally, as the perceived benefits of blue-blocking functionality are not immediately noticeable in daily life, patients may prioritize other, more tangible features, such as presbyopia or spherical aberration correction.

Similarly, although the surgeon’s recommendation was included as an attribute, it did not significantly influence patient preferences. This suggests that patients in this study may place more value on the specific features of the IOLs rather than the recommendation of the surgeon, or it may reflect the growing trend of patients actively researching and making informed decisions about their healthcare. However, this finding also highlights the need for healthcare professionals to provide detailed information and personalized advice to patients, ensuring that recommendations are based on individual needs and preferences.

### 4.5 Preference heterogeneity and demographic insights

Significant heterogeneity in patient preferences was observed, particularly with respect to gender and education level. Men, for instance, were more likely to prefer toric IOLs, potentially due to their higher spatial and temporal acuity (20) and greater openness to advanced technologies (21). This preference aligns with studies suggesting that men often exhibit superior visual performance and may value the sharpness and clarity provided by toric IOLs. Additionally, cost-sensitivity was more pronounced in men, with a higher proportion of males in the “Astigmatism and Cost-Sensitive Patients” subgroup. These insights provide valuable guidance for tailoring IOLs selection to gender-specific preferences, with manufacturers potentially focusing on cost-effective, multi-functional lenses that correct astigmatism for this group.

Education level also played a significant role in shaping preferences. Patients with higher education levels were less inclined to choose multifocal IOLs, which contrasts with traditional assumptions that multifocal lenses are primarily favored by more educated individuals. This finding challenges the conventional view and suggests that multifocal IOLs may now appeal to a broader range of patients, driven by the increasing demand for near-vision correction due to lifestyle changes, such as the widespread use of digital devices.

### 4.6 Study limitations and future directions

This study has several limitations that should be addressed in future research. First, reliance on self-reported preferences via a questionnaire may introduce subjective biases, and the DCE method, while valuable, treats each attribute independently, potentially overlooking interactions between features. Furthermore, the study did not compare questionnaire-based preferences with actual surgical decisions, limiting its real-world applicability. Future studies should aim to bridge this gap by comparing stated preferences with actual choices made in clinical settings.

Additionally, while the DCE method identifies preference patterns, it does not uncover the underlying cognitive or emotional factors driving these choices. Future research could benefit from qualitative methods, such as interviews or focus groups, to gain deeper insights into the psychological factors that influence decision-making. This would complement the DCE findings and provide a more holistic understanding of patient preferences, enhancing the relevance of these studies for clinical practice and policy development.

## 5 Conclusion

In conclusion, this study offers important insights into cataract patients’ preferences for IOLs, highlighting cost, presbyopia correction, aberration correction, and astigmatism correction as the primary factors shaping patient choices. The results underscore the need for healthcare policies that address financial barriers to IOLs selection and promote the availability of advanced IOLs options. Additionally, preference heterogeneity based on gender and education level provides valuable guidance for personalized decision-making in cataract surgery. This study lays the foundation for future research aimed at further understanding patient preferences and improving shared decision-making in cataract treatment.

## Data Availability

The raw data supporting the conclusions of this article will be made available by the authors, without undue reservation.
