# Supplementary material for: Exploring patient preferences for intraocular lenses selection in age-related cataract: a discrete choice experiment study
Source: Front Med (Lausanne). 2025 Feb 12;12:1446715. doi: 10.3389/fmed.2025.1446715 (PMC11861010; doi:10.3389/fmed.2025.1446715)
Supplement: Supplementary file 1 [file Data_Sheet_1.pdf]

BLOCK ONE

Please compare the IOL offered, and tick which IOL you would choose

	OPTION A	OPTION B
Blue-blocking	YES<br>Blue light (with potential harm to the retina) is blocked	NO<br>Blue light (with potential harm to the retina) is not blocked
Spherical aberration correction	NO<br>Spherical aberration, causing distortion in peripheral vision, is not corrected	YES<br>Spherical aberration, causing distortion in peripheral vision, is corrected
Astigmatism correction	NO<br>Astigmatism (resulting in clarity on certain axes and blurriness on others) is not corrected	YES<br>Astigmatism (resulting in clarity on certain axes and blurriness on others) is corrected
Presbyopia correction	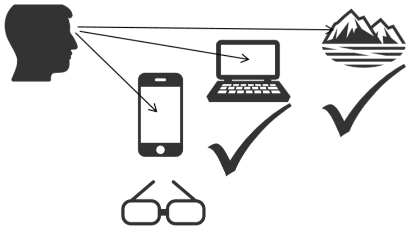	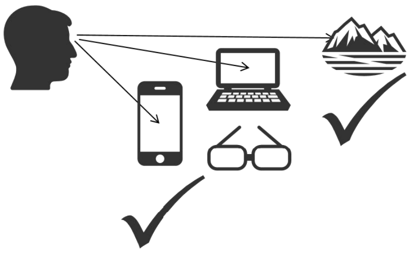
Probability of adverse visual phenomena	Risk of undesired visual effects (such as halos, glare, and starbursts)<br>10%	Risk of undesired visual effects (such as halos, glare, and starbursts)<br>20%
Recommendation from surgeon	WITH	WITHOUT
Cost	CNY ¥ 6000	CNY ¥ 10000

	OPTION A	OPTION B
Blue-blocking	YES<br>Blue light (with potential harm to the retina) is blocked	NO<br>Blue light (with potential harm to the retina) is not blocked
Spherical aberration correction	YES<br>Spherical aberration, causing distortion in peripheral vision, is corrected	NO<br>Spherical aberration, causing distortion in peripheral vision, is not corrected
Astigmatism correction	YES<br>Astigmatism (resulting in clarity on certain axes and blurriness on others) is corrected	NO<br>Astigmatism (resulting in clarity on certain axes and blurriness on others) is not corrected
Presbyopia correction	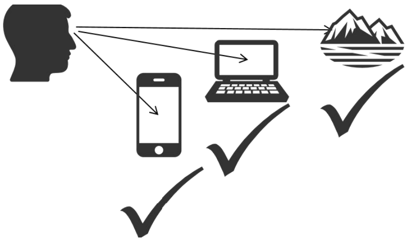	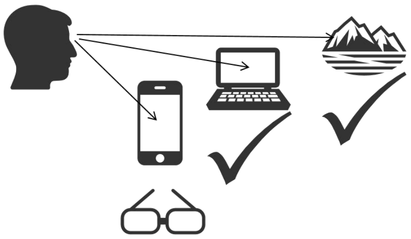
Probability of adverse visual phenomena	Risk of undesired visual effects (such as halos, glare, and starbursts)<br>0%	Risk of undesired visual effects (such as halos, glare, and starbursts)<br>10%
Recommendation from surgeon	WITH	WITHOUT
Cost	CNY ¥ 30000	CNY ¥ 10000

	OPTION A	OPTION B
Blue-blocking	NO<br>Blue light (with potential harm to the retina) is not blocked	YES<br>Blue light (with potential harm to the retina) is blocked
Spherical aberration correction	YES<br>Spherical aberration, causing distortion in peripheral vision, is corrected	NO<br>Spherical aberration, causing distortion in peripheral vision, is not corrected
Astigmatism correction	YES<br>Astigmatism (resulting in clarity on certain axes and blurriness on others) is corrected	NO<br>Astigmatism (resulting in clarity on certain axes and blurriness on others) is not corrected
Presbyopia correction	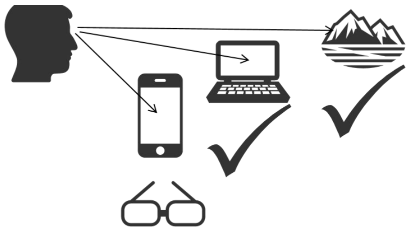	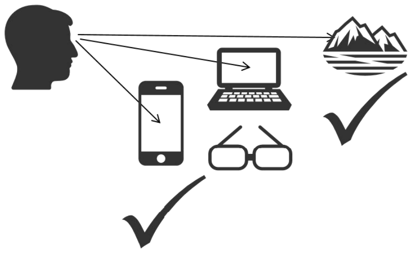
Probability of adverse visual phenomena	Risk of undesired visual effects (such as halos, glare, and starbursts)<br>0%	Risk of undesired visual effects (such as halos, glare, and starbursts)<br>20%
Recommendation from surgeon	WITHOUT	WITH
Cost	CNY ¥ 6000	CNY ¥ 3000

	OPTION A	OPTION B
Blue-blocking	NO<br>Blue light (with potential harm to the retina) is not blocked	YES<br>Blue light (with potential harm to the retina) is blocked
Spherical aberration correction	YES<br>Spherical aberration, causing distortion in peripheral vision, is corrected	NO<br>Spherical aberration, causing distortion in peripheral vision, is not corrected
Astigmatism correction	NO<br>Astigmatism (resulting in clarity on certain axes and blurriness on others) is not corrected	YES<br>Astigmatism (resulting in clarity on certain axes and blurriness on others) is corrected
Presbyopia correction	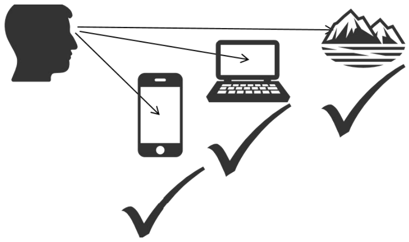	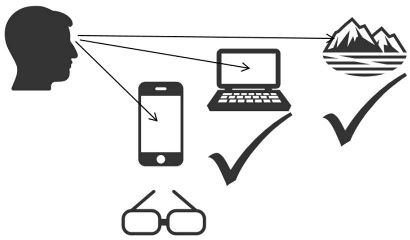
Probability of adverse visual phenomena	Risk of undesired visual effects (such as halos, glare, and starbursts)<br>40%	Risk of undesired visual effects (such as halos, glare, and starbursts)<br>0%
Recommendation from surgeon	WITH	WITHOUT
Cost	CNY ¥ 10000	CNY ¥ 3000

Repeated task

	OPTION A	OPTION B
Blue-blocking	YES<br>Blue light (with potential harm to the retina) is blocked	NO<br>Blue light (with potential harm to the retina) is not blocked
Spherical aberration correction	NO<br>Spherical aberration, causing distortion in peripheral vision, is not corrected	YES<br>Spherical aberration, causing distortion in peripheral vision, is corrected
Astigmatism correction	NO<br>Astigmatism (resulting in clarity on certain axes and blurriness on others) is not corrected	YES<br>Astigmatism (resulting in clarity on certain axes and blurriness on others) is corrected
Presbyopia correction	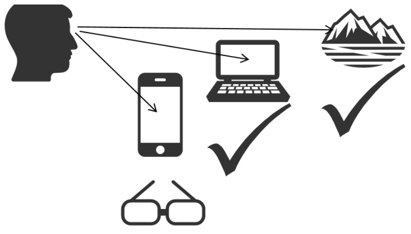	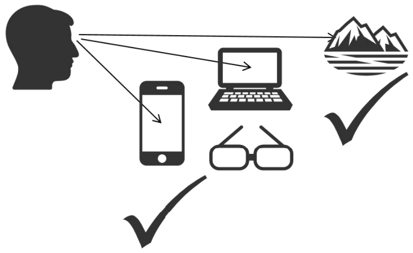
Probability of adverse visual phenomena	Risk of undesired visual effects (such as halos, glare, and starbursts)<br>10%	Risk of undesired visual effects (such as halos, glare, and starbursts)<br>20%
Recommendation from surgeon	WITH	WITHOUT
Cost	CNY ¥ 6000	CNY ¥ 10000

	OPTION A	OPTION B
Blue-blocking	YES<br>Blue light (with potential harm to the retina) is blocked	NO<br>Blue light (with potential harm to the retina) is not blocked
Spherical aberration correction	NO<br>Spherical aberration, causing distortion in peripheral vision, is not corrected	YES<br>Spherical aberration, causing distortion in peripheral vision, is corrected
Astigmatism correction	NO<br>Astigmatism (resulting in clarity on certain axes and blurriness on others) is not corrected	YES<br>Astigmatism (resulting in clarity on certain axes and blurriness on others) is corrected
Presbyopia correction	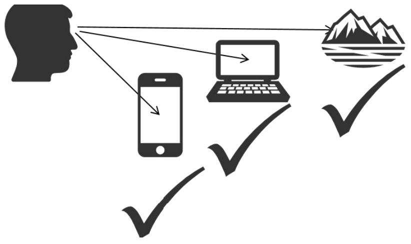	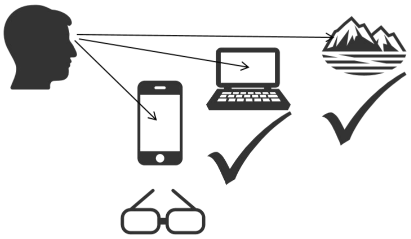
Probability of adverse visual phenomena	Risk of undesired visual effects (such as halos, glare, and starbursts)<br>0%	Risk of undesired visual effects (such as halos, glare, and starbursts)<br>20%
Recommendation from surgeon	WITHOUT	WITH
Cost	CNY ¥ 10000	CNY ¥ 3000

	OPTION A	OPTION B
Blue-blocking	NO<br>Blue light (with potential harm to the retina) is not blocked	YES<br>Blue light (with potential harm to the retina) is blocked
Spherical aberration correction	YES<br>Spherical aberration, causing distortion in peripheral vision, is corrected	NO<br>Spherical aberration, causing distortion in peripheral vision, is not corrected
Astigmatism correction	NO<br>Astigmatism (resulting in clarity on certain axes and blurriness on others) is not corrected	YES<br>Astigmatism (resulting in clarity on certain axes and blurriness on others) is corrected
Presbyopia correction	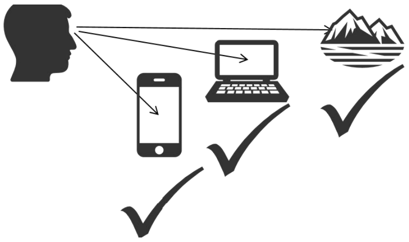	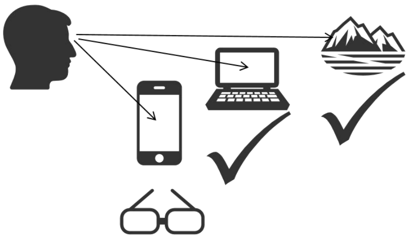
Probability of adverse visual phenomena	Risk of undesired visual effects (such as halos, glare, and starbursts)<br>30%	Risk of undesired visual effects (such as halos, glare, and starbursts)<br>40%
Recommendation from surgeon	WITH	WITHOUT
Cost	CNY ¥ 6000	CNY ¥ 2000

	OPTION A	OPTION B
Blue-blocking	YES<br>Blue light (with potential harm to the retina) is blocked	NO<br>Blue light (with potential harm to the retina) is not blocked
Spherical aberration correction	NO<br>Spherical aberration, causing distortion in peripheral vision, is not corrected	YES<br>Spherical aberration, causing distortion in peripheral vision, is corrected
Astigmatism correction	YES<br>Astigmatism (resulting in clarity on certain axes and blurriness on others) is corrected	NO<br>Astigmatism (resulting in clarity on certain axes and blurriness on others) is not corrected
Presbyopia correction	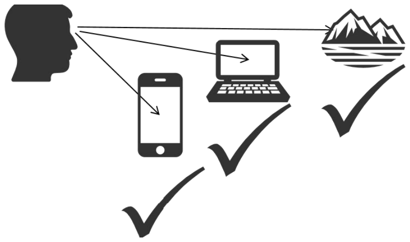	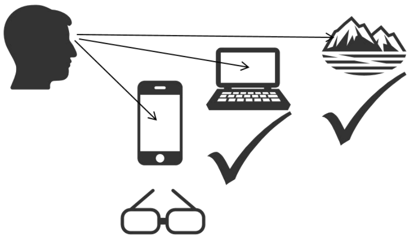
Probability of adverse visual phenomena	Risk of undesired visual effects (such as halos, glare, and starbursts)<br>20%	Risk of undesired visual effects (such as halos, glare, and starbursts)<br>0%
Recommendation from surgeon	WITHOUT	WITH
Cost	CNY ¥ 6000	CNY ¥ 3000

	OPTION A	OPTION B
Blue-blocking	NO<br>Blue light (with potential harm to the retina) is not blocked	YES<br>Blue light (with potential harm to the retina) is blocked
Spherical aberration correction	NO<br>Spherical aberration, causing distortion in peripheral vision, is not corrected	YES<br>Spherical aberration, causing distortion in peripheral vision, is corrected
Astigmatism correction	YES<br>Astigmatism (resulting in clarity on certain axes and blurriness on others) is corrected	NO<br>Astigmatism (resulting in clarity on certain axes and blurriness on others) is not corrected
Presbyopia correction	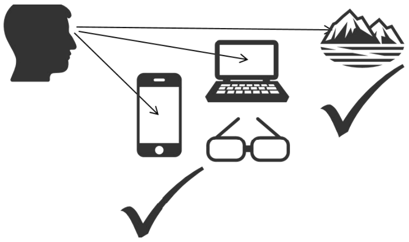	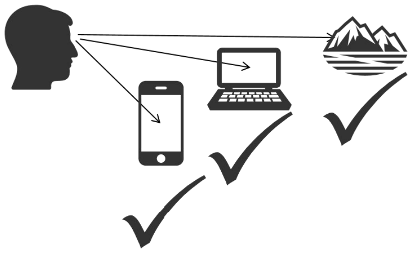
Probability of adverse visual phenomena	Risk of undesired visual effects (such as halos, glare, and starbursts)<br>0%	Risk of undesired visual effects (such as halos, glare, and starbursts)<br>30%
Recommendation from surgeon	WITH	WITHOUT
Cost	CNY ¥ 6000	CNY ¥ 2000

Dominance task

	OPTION A	OPTION B
Blue-blocking	NO<br>Blue light (with potential harm to the retina) is not blocked	YES<br>Blue light (with potential harm to the retina) is blocked
Spherical aberration correction	NO<br>Spherical aberration, causing distortion in peripheral vision, is not corrected	YES<br>Spherical aberration, causing distortion in peripheral vision, is corrected
Astigmatism correction	NO<br>Astigmatism (resulting in clarity on certain axes and blurriness on others) is not corrected	YES<br>Astigmatism (resulting in clarity on certain axes and blurriness on others) is corrected
Presbyopia correction	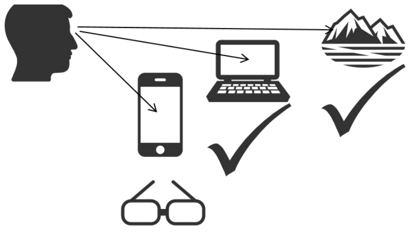	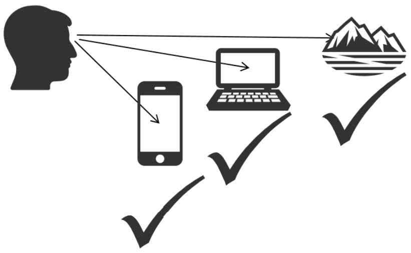
Probability of adverse visual phenomena	Risk of undesired visual effects (such as halos, glare, and starbursts)<br>40%	Risk of undesired visual effects (such as halos, glare, and starbursts)<br>0%
Recommendation from surgeon	WITHOUT	WITH
Cost	CNY ¥ 30000	CNY ¥ 2000

BLOCK TWO

Please compare the IOL offered, and tick which IOL you would choose

	OPTION A	OPTION B
Blue-blocking	YES<br>Blue light (with potential harm to the retina) is blocked	NO<br>Blue light (with potential harm to the retina) is not blocked
Spherical aberration correction	YES<br>Spherical aberration, causing distortion in peripheral vision, is corrected	NO<br>Spherical aberration, causing distortion in peripheral vision, is not corrected
Astigmatism correction	NO<br>Astigmatism (resulting in clarity on certain axes and blurriness on others) is not corrected	YES<br>Astigmatism (resulting in clarity on certain axes and blurriness on others) is corrected
Presbyopia correction	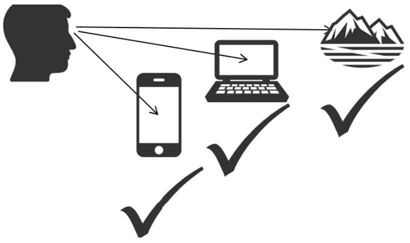	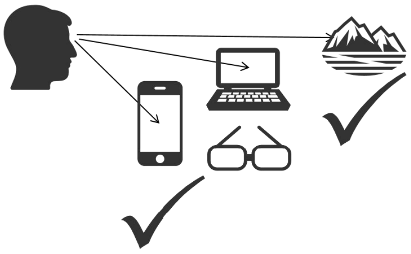
Probability of adverse visual phenomena	Risk of undesired visual effects (such as halos, glare, and starbursts)<br>10%	Risk of undesired visual effects (such as halos, glare, and starbursts)<br>30%
Recommendation from surgeon	WITHOUT	WITH
Cost	CNY ¥ 3000	CNY ¥ 2000

	OPTION A	OPTION B
Blue-blocking	YES<br>Blue light (with potential harm to the retina) is blocked	NO<br>Blue light (with potential harm to the retina) is not blocked
Spherical aberration correction	YES<br>Spherical aberration, causing distortion in peripheral vision, is corrected	NO<br>Spherical aberration, causing distortion in peripheral vision, is not corrected
Astigmatism correction	YES<br>Astigmatism (resulting in clarity on certain axes and blurriness on others) is corrected	NO<br>Astigmatism (resulting in clarity on certain axes and blurriness on others) is not corrected
Presbyopia correction	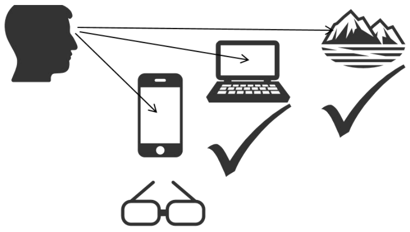	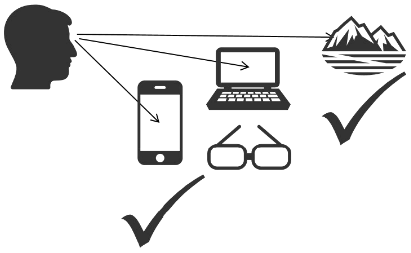
Probability of adverse visual phenomena	Risk of undesired visual effects (such as halos, glare, and starbursts)<br>30%	Risk of undesired visual effects (such as halos, glare, and starbursts)<br>20%
Recommendation from surgeon	WITH	WITHOUT
Cost	CNY ¥ 10000	CNY ¥ 30000

	OPTION A	OPTION B
Blue-blocking	NO<br>Blue light (with potential harm to the retina) is not blocked	YES<br>Blue light (with potential harm to the retina) is blocked
Spherical aberration correction	YES<br>Spherical aberration, causing distortion in peripheral vision, is corrected	NO<br>Spherical aberration, causing distortion in peripheral vision, is not corrected
Astigmatism correction	NO<br>Astigmatism (resulting in clarity on certain axes and blurriness on others) is not corrected	YES<br>Astigmatism (resulting in clarity on certain axes and blurriness on others) is corrected
Presbyopia correction	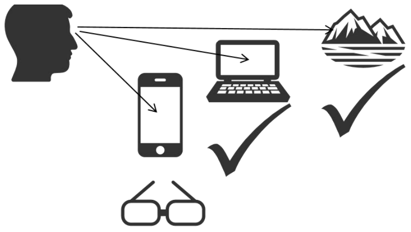	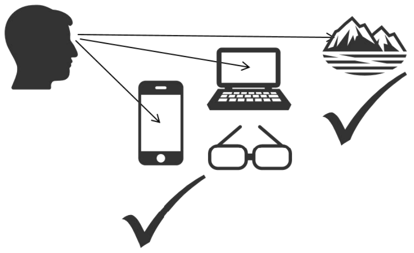
Probability of adverse visual phenomena	Risk of undesired visual effects (such as halos, glare, and starbursts)<br>20%	Risk of undesired visual effects (such as halos, glare, and starbursts)<br>40%
Recommendation from surgeon	WITHOUT	WITH
Cost	CNY ¥ 2000	CNY ¥ 3000

	OPTION A	OPTION B

Blue-blocking	NO<br>Blue light (with potential harm to the retina) is not blocked	YES<br>Blue light (with potential harm to the retina) is blocked
Spherical aberration correction	NO<br>Spherical aberration, causing distortion in peripheral vision, is not corrected	YES<br>Spherical aberration, causing distortion in peripheral vision, is corrected
Astigmatism correction	YES<br>Astigmatism (resulting in clarity on certain axes and blurriness on others) is corrected	NO<br>Astigmatism (resulting in clarity on certain axes and blurriness on others) is not corrected
Presbyopia correction	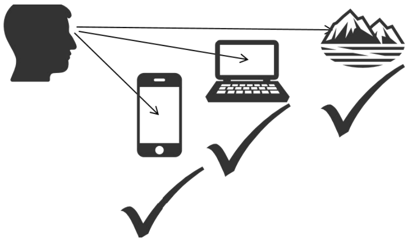	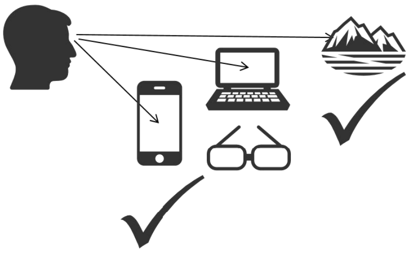
Probability of adverse visual phenomena	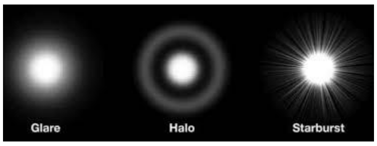<br>10%	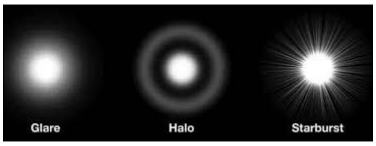<br>30%
Recommendation from surgeon	WITH	WITHOUT
Cost	CNY ¥ 2000	CNY ¥ 6000

Repeated task

	OPTION A	OPTION B
Blue-blocking	YES<br>Blue light (with potential harm to the retina) is blocked	NO<br>Blue light (with potential harm to the retina) is not blocked
Spherical aberration correction	YES<br>Spherical aberration, causing distortion in peripheral vision, is corrected	NO<br>Spherical aberration, causing distortion in peripheral vision, is not corrected
Astigmatism correction	NO<br>Astigmatism (resulting in clarity on certain axes and blurriness on others) is not corrected	YES<br>Astigmatism (resulting in clarity on certain axes and blurriness on others) is corrected
Presbyopia correction	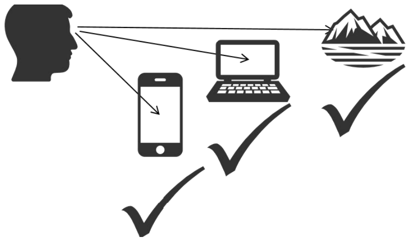	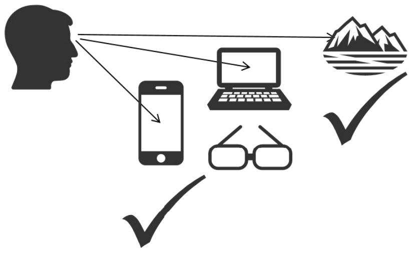
Probability of adverse visual phenomena	Risk of undesired visual effects (such as halos, glare, and starbursts)<br>10%	Risk of undesired visual effects (such as halos, glare, and starbursts)<br>30%
Recommendation from surgeon	WITHOUT	WITH
Cost	CNY ¥ 3000	CNY ¥ 2000

	OPTION A	OPTION B
Blue-blocking	YES<br>Blue light (with potential harm to the retina) is blocked	NO<br>Blue light (with potential harm to the retina) is not blocked
Spherical aberration correction	YES<br>Spherical aberration, causing distortion in peripheral vision, is corrected	NO<br>Spherical aberration, causing distortion in peripheral vision, is not corrected
Astigmatism correction	NO<br>Astigmatism (resulting in clarity on certain axes and blurriness on others) is not corrected	YES<br>Astigmatism (resulting in clarity on certain axes and blurriness on others) is corrected
Presbyopia correction	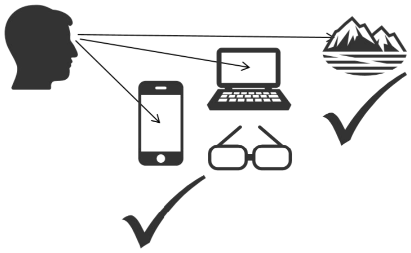	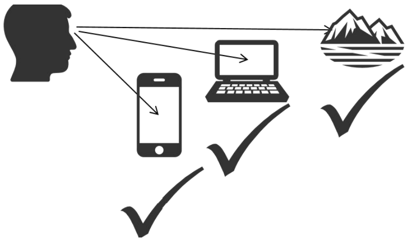
Probability of adverse visual phenomena	Risk of undesired visual effects (such as halos, glare, and starbursts)<br>10%	Risk of undesired visual effects (such as halos, glare, and starbursts)<br>30%
Recommendation from surgeon	WITH	WITHOUT
Cost	CNY ¥ 2000	CNY ¥ 3000

	OPTION A	OPTION B
Blue-blocking	NO<br>Blue light (with potential harm to the retina) is not blocked	YES<br>Blue light (with potential harm to the retina) is blocked
Spherical aberration correction	YES<br>Spherical aberration, causing distortion in peripheral vision, is corrected	NO<br>Spherical aberration, causing distortion in peripheral vision, is not corrected
Astigmatism correction	YES<br>Astigmatism (resulting in clarity on certain axes and blurriness on others) is corrected	NO<br>Astigmatism (resulting in clarity on certain axes and blurriness on others) is not corrected
Presbyopia correction	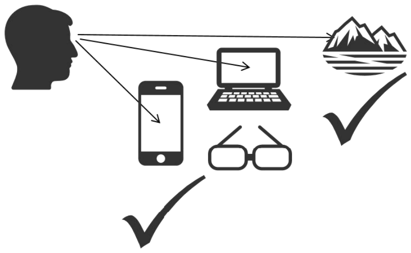	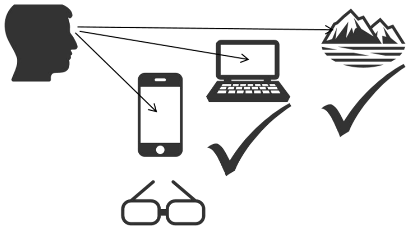
Probability of adverse visual phenomena	Risk of undesired visual effects (such as halos, glare, and starbursts)<br>10%	Risk of undesired visual effects (such as halos, glare, and starbursts)<br>20%
Recommendation from surgeon	WITHOUT	WITH
Cost	CNY ¥ 30000	CNY ¥ 10000

	OPTION A	OPTION B
Blue-blocking	YES<br>Blue light (with potential harm to the retina) is blocked	NO<br>Blue light (with potential harm to the retina) is not blocked
Spherical aberration correction	NO<br>Spherical aberration, causing distortion in peripheral vision, is not corrected	YES<br>Spherical aberration, causing distortion in peripheral vision, is corrected
Astigmatism correction	YES<br>Astigmatism (resulting in clarity on certain axes and blurriness on others) is corrected	NO<br>Astigmatism (resulting in clarity on certain axes and blurriness on others) is not corrected
Presbyopia correction	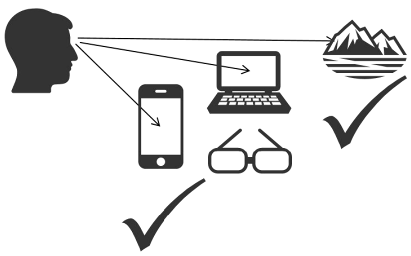	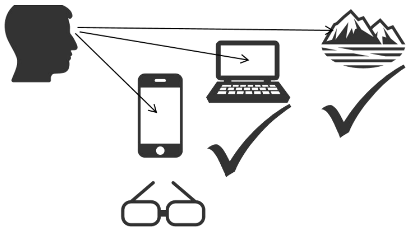
Probability of adverse visual phenomena	Risk of undesired visual effects (such as halos, glare, and starbursts)<br>10%	Risk of undesired visual effects (such as halos, glare, and starbursts)<br>40%
Recommendation from surgeon	WITH	WITHOUT
Cost	CNY ¥ 10000	CNY ¥ 6000

	OPTION A	OPTION B
Blue-blocking	YES<br>Blue light (with potential harm to the retina) is blocked	NO<br>Blue light (with potential harm to the retina) is not blocked
Spherical aberration correction	YES<br>Spherical aberration, causing distortion in peripheral vision, is corrected	NO<br>Spherical aberration, causing distortion in peripheral vision, is not corrected
Astigmatism correction	YES<br>Astigmatism (resulting in clarity on certain axes and blurriness on others) is corrected	NO<br>Astigmatism (resulting in clarity on certain axes and blurriness on others) is not corrected
Presbyopia correction	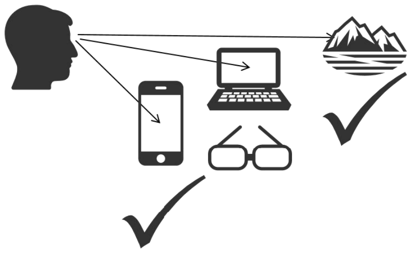	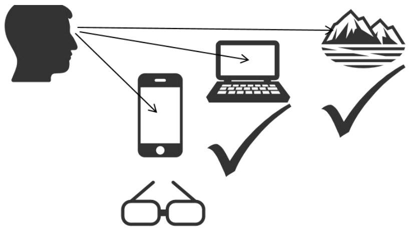
Probability of adverse visual phenomena	Risk of undesired visual effects (such as halos, glare, and starbursts)<br>20%	Risk of undesired visual effects (such as halos, glare, and starbursts)<br>30%
Recommendation from surgeon	WITHOUT	WITH
Cost	CNY ¥ 10000	CNY ¥ 30000

Dominance task

	OPTION A	OPTION B
Blue-blocking	NO<br>Blue light (with potential harm to the retina) is not blocked	YES<br>Blue light (with potential harm to the retina) is blocked
Spherical aberration correction	NO<br>Spherical aberration, causing distortion in peripheral vision, is not corrected	YES<br>Spherical aberration, causing distortion in peripheral vision, is corrected
Astigmatism correction	NO<br>Astigmatism (resulting in clarity on certain axes and blurriness on others) is not corrected	YES<br>Astigmatism (resulting in clarity on certain axes and blurriness on others) is corrected
Presbyopia correction	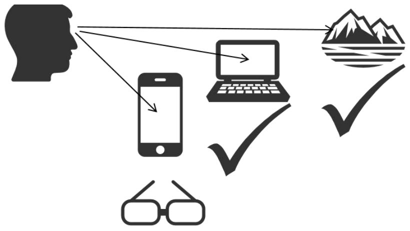	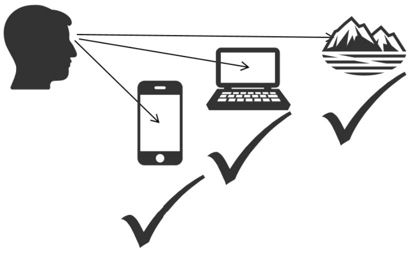
Probability of adverse visual phenomena	Risk of undesired visual effects (such as halos, glare, and starbursts)<br>40%	Risk of undesired visual effects (such as halos, glare, and starbursts)<br>0%
Recommendation from surgeon	WITHOUT	WITH
Cost	CNY ¥ 30000	CNY ¥ 2000
